# Anatomical variation in the ankle and foot: from incidental finding to inductor of pathology. Part II: midfooot and forefoot

**DOI:** 10.1186/s13244-019-0747-1

**Published:** 2019-07-31

**Authors:** Maria Pilar Aparisi Gómez, Francisco Aparisi, Alessandra Bartoloni, Maria Alejandra Ferrando Fons, Giuseppe Battista, Giuseppe Guglielmi, Alberto Bazzocchi

**Affiliations:** 1 Department of Radiology, Auckland City Hospital - Auckland District Health Board (ADHB), 2 Park Road, Grafton, Auckland, 1023 New Zealand; 2 Department of Radiology, Hospital Vithas Nueve de Octubre, Calle Valle de la Ballestera, 59, 46015 Valencia, Spain; 3 Department of Diagnostic Imaging, Bambino Gesù Children Hospital, Piazza Sant’Onofrio 4, 00165 Rome, Italy; 4 Department of Orthopaedics and Traumatology, Malteser Krankenhaus St. Josefshospital, Kurfürstenstrasse 69, 47829 Krefeld, Germany; 5 Department of Experimental, Diagnostic and Specialty Medicine (DIMES). University of Bologna, S.Orsola-Malpighi Hospital, Via G. Massarenti 9, 40138 Bologna, Italy; 6 Department of Radiology, University of Foggia, Viale Luigi Pinto 1, 71100 Foggia, Italy; 7 Diagnostic and Interventional Radiology, IRCCS Istituto Ortopedico Rizzoli, Via G. C. Pupilli 1, 40136 Bologna, Italy

**Keywords:** Foot, Accessory ossicles, Accessory muscles, Computed tomography, Magnetic resonance

## Abstract

Accessory anatomical structures in the ankle and foot usually represent incidental imaging findings; however, they may also eventually represent a source of pathology, such as painful syndromes, degenerative changes, be the subject of overuse and trauma, or appear as masses and cause compression syndromes or impingement. This review aims to describe and illustrate the imaging findings related to the presence of accessory ossicles and muscles in the midfoot and forefoot through different techniques, with special attention on those variants that associate factors of clinical relevance or that would trigger challenges in the differential diagnosis.

## Key points

- Accessory anatomical structures in midfoot and forefoot are a common incidental finding
- Anatomical variants may trigger challenges in the differential diagnosis
- Anatomical variants may be a source of pathology

## Introduction

A number of anatomical variations can be found in the ankle and foot. These include accessory ossicles, additional sesamoid bones, variations in number and configuration of sesamoid bones, coalitions, bipartitions, and variants in the soft tissues, such as accessory muscles.

These findings are subject to a lot of variation. Most of them represent developmental abnormalities that constitute incidental radiographic findings [1].

Accessory ossicles in most cases are a result of unfused ossification centers. They are seen as subdivisions of existing bones or free elements in vicinity of the normal bone structures. Sesamoid bones have a different anatomical nature. They functionally represent components of a gliding mechanism and are at least partially embedded in tendons, reducing friction and protecting the tendon structure [1, 2].

The most common accessory ossicles in the ankle and foot are the os trigonum, the accessory navicular (among the different three types, type II is the most common), and the os intermetatarseum, in this order. Regarding accessory sesamoid bones, the os peroneum is the most frequently found [2].

Accessory ossicles and muscles are also generally asymptomatic, and discovered incidentally on imaging studies. However, they may also eventually represent a source of pathology, giving rise to painful syndromes, degenerative changes, be the subject of overuse and trauma, or appear as masses and cause compression syndromes or impingement.

Our aim with this review is to illustrate the imaging findings related to the presence of accessory ossicles and muscles in the ankle and foot through different techniques, with special attention to those variants that associate factors of clinical relevance or, in the case of the ossicles, would pose a challenge in the differential with fractures.

Bone coalitions, given their complexity and frequent clinical implications, deserve separate analysis and will not be the object of this review.

## Midfoot

The midfoot is defined as the region in between the Chopart joint (talo-navicular and calcaneo-cuboid joints) and the Lisfranc joint (tarso-metatarsal joint)

**Fig. 1:**
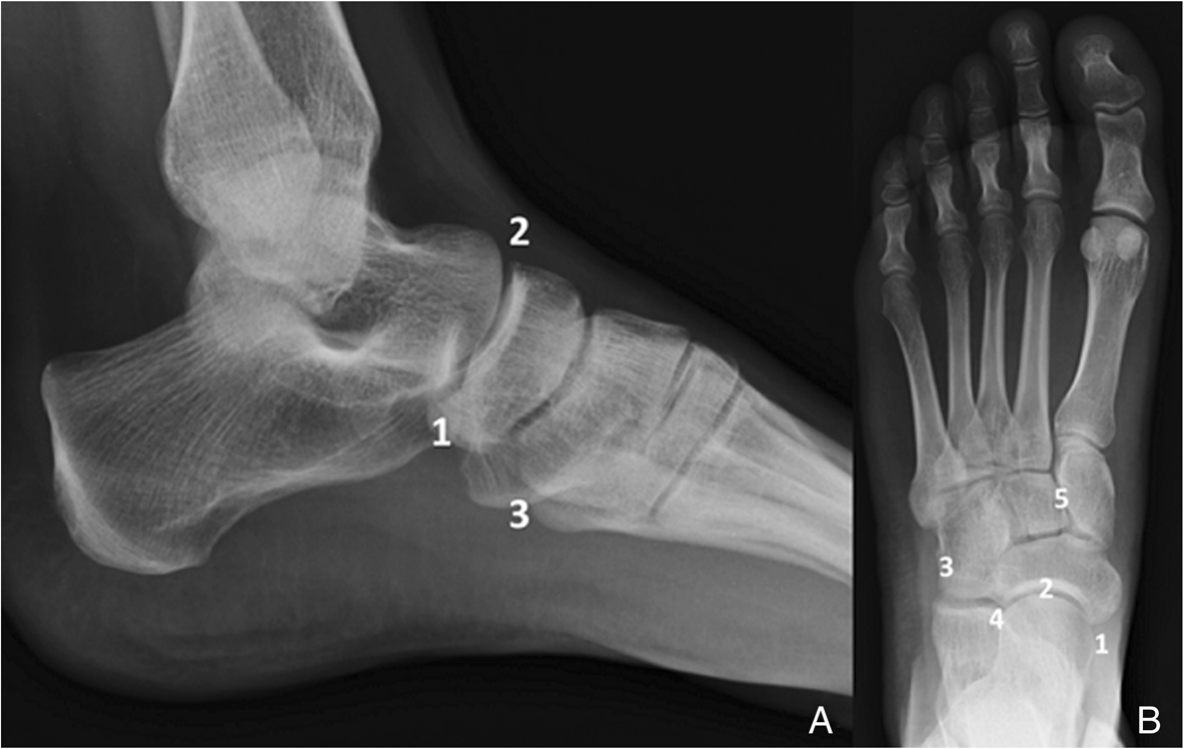
Diagram of the location of the most common accessory bones of the midfoot. **a** Lateral and (**b**) AP projection of the midfoot. 1—Accessory navicular (different types), 2—os supranaviculare, 3—os peroneum (sesamoid), 4—os cuboideum secundarium, 5—os intercuneiform

- Ossicles (Fig. 1) (Table 1)

**Table 1:** Prevalence, clinical significance, and differential diagnosis of the most common types of variants and accessory ossicles in the midfoot

Ossicle		Prevalence			Clinical significance	Differential diagnosis
Accessory navicular		4–21%	I	~ 30%	Asymptomatic	–
			II	50% bilateral 50–90%	Disruption of the synchondrosis Chronic tendinosis or tear Flat-foot deformity Osteonecrosis	Avulsion fractures of the tuberosity
			III	~ 30%	Irritation of the surrounding tissues Adventitial bursa formation Flat foot deformity	–
Os supranaviculare		1%			Asymptomatic	Avulsion fractures of the capsule of the talonavicular joint
Os peroneum		3–26% (ossified form) multipartite – 30% bilateral – 60%			Painful os peroneum syndrome Peroneus longus tear	Fracture (os trigonum—os subfibulare if migrated, multipartite if not)
Rare	Bipartite medial cuneiform	0.3–2.4%			Degeneration and overuse syndromes	Fracture
	Os cuboideum secundarium	–			Asymptomatic	Potential to mimic a mass on MR
	Os intercuneiforme	0.026%			Asymptomatic	Fracture

### Accessory navicular

The accessory navicular is also known as the os tibiale, os tibiale externum, or naviculare secundarium.

Estimated prevalence has been set in between 4 and 21% [1–3]. A recent long series by Kalbouneh et al. estimates it as 20.9% [4].

The accessory navicular is located adjacent to the postero-medial tuberosity of the bone, and three different configurations exist [5].

The type I is an oval or round small ossified structure, located within the tibialis posterior tendon. In some cases, there are several of them (multiple configuration) [5].

**Fig. 2:**
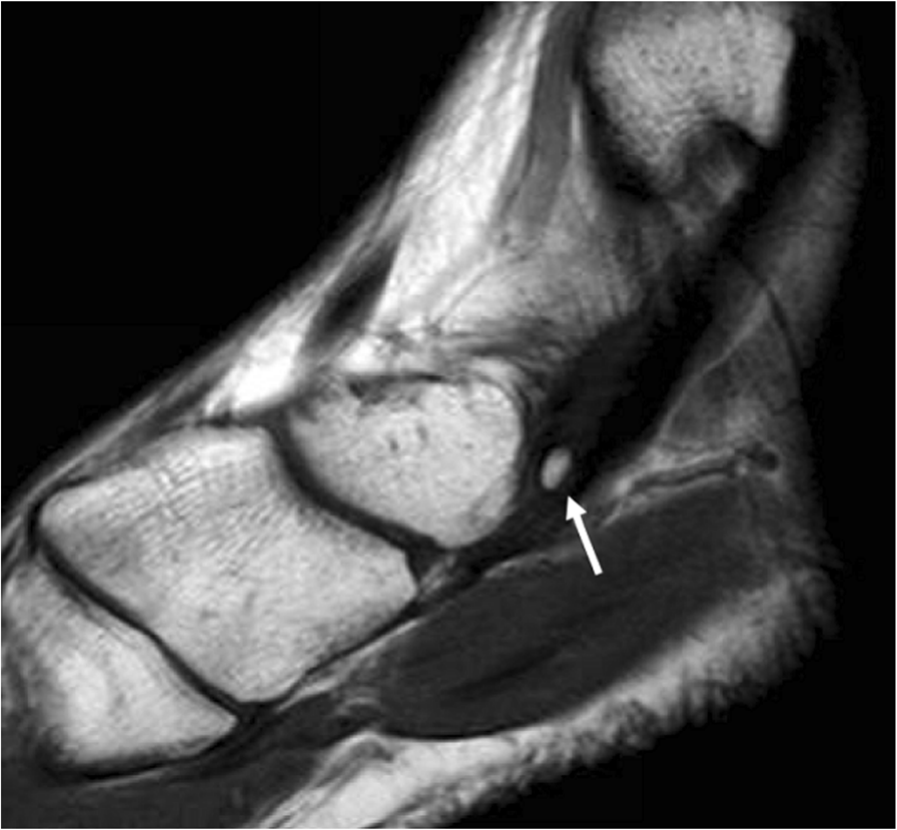
Type I accessory navicular. Sagittal fast spin echo T1 (FSE T1) demonstrates the incidental finding of a type I accessory navicular in a 33-year-old man, referred with the suspicion of arthropathy. Signal intensity is normal. Note how this is embedded in the tibialis posterior tendon (white arrow)

They may be seen separated to up to 5 mm from the navicular tuberosity. Its prevalence is approximately 30% [6] and represents the classically known os tibiale externum. Type I accessory navicular are usually asymptomatic **(**Fig. 2**)**. Interestingly, the os tibiale externum has been commonly described as a sesamoid in the literature [3–7], which represents a different concept from an accessory ossicle. The distinction between the cases in which this represents a true accessory ossicle versus a sesamoid has been made in the literature, in a cadaveric study by Bareither et al. They demonstrated that when this structure is separated 3 mm or more from the tuberosity, there is no fibrotic connection to the navicular tuberosity and therefore the finding can be considered a sesamoid. When the bony structure is located at a distance of less than 3 mm, there is normally a fibrotic attachment to the navicular tuberosity, and thus represents a true accessory ossicle [8].

Type II is the most prevalent, up to 50% [9] of accessory navicular and one of the most prevalent accessory bones in the foot, with an estimated total prevalence of 2–12% [1–3,7,9]. Type II consists of a triangular or hemispherical unfused accessory ossification center, separated from the navicular tubercle by a 1–2 mm synchondrosis. It is also called os naviculare. Can be bilateral in 50 to 90% of the cases [10]. This type of accessory navicular is the most commonly symptomatic one.

**Fig. 3:**
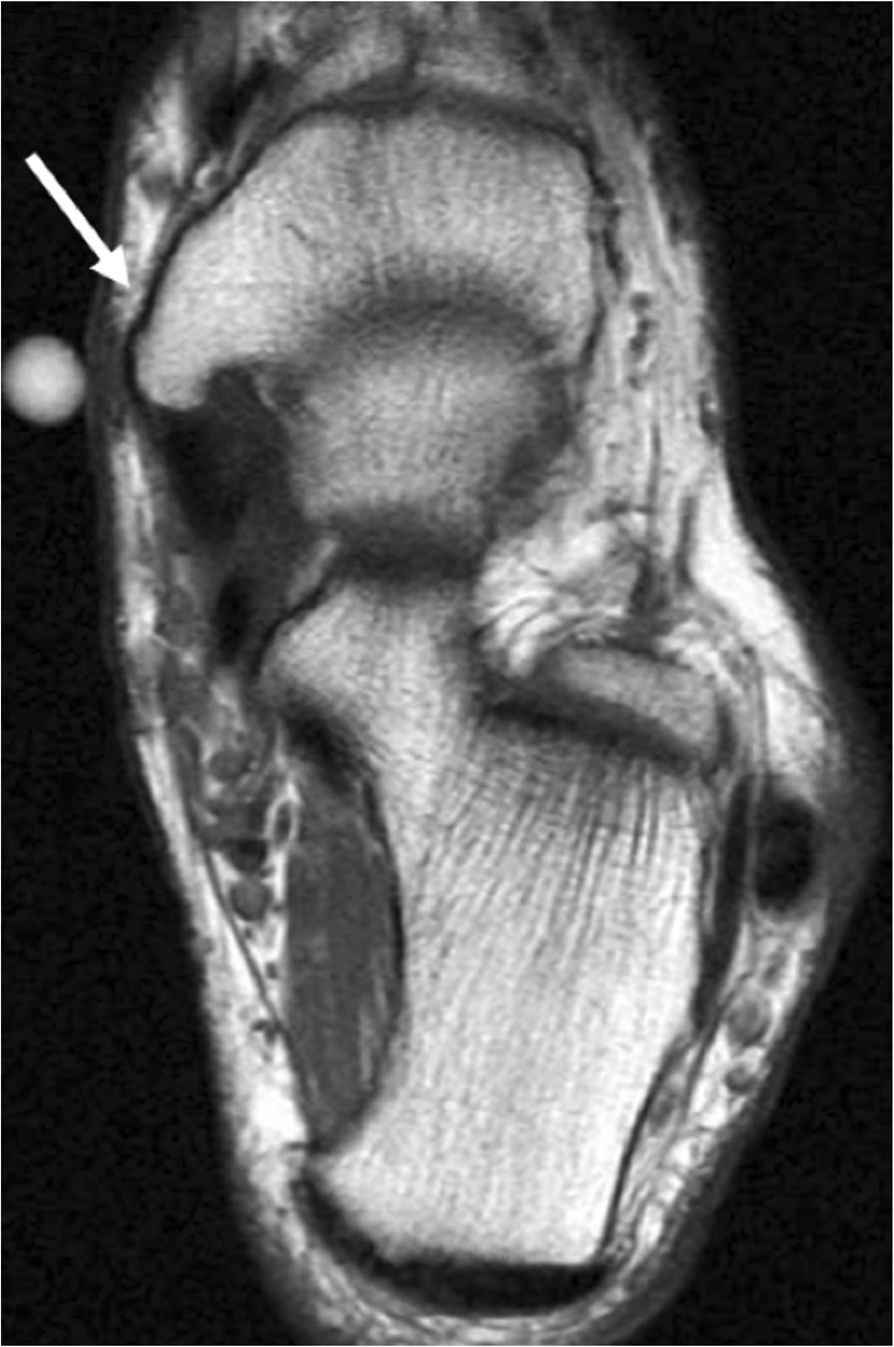
Type III accessory navicular. Axial FSE T1 on a 60-year-old man with pain in the medial aspect of the foot, over the bony prominence of a type III accessory navicular (white arrow). Note a region of decreased signal and trabeculation of the subcutaneous fat over the prominence of the tuberosity (vitamin A marker). This suggests a degree of irritation of the surrounding tissues due to friction

Type III consists of a prominent tuberosity, and is the least frequent, with an estimated prevalence of 30% among accessory navicular [6]. It is also called cornuate navicular. Many authors actually consider it a type II that has fused to the tubercle. It may become symptomatic by irritation of the surrounding tissues, with possible adventitial bursa formation [10] and flat foot deformity [4] (Fig. 3).

Symptomatic accessory navicular is most commonly seen in the cases of type II accessory navicular. This is mainly the result of altered biomechanics.

When this accessory bone is present, the distal portion of the tibialis posterior tendon straightens, causing adduction forces that can result in flat-foot deformity, but besides, the tendon can be repeatedly impinged on dorsiflexion of the ankle, which may result in chronic tendinosis or tear [9].

**Fig. 4:**
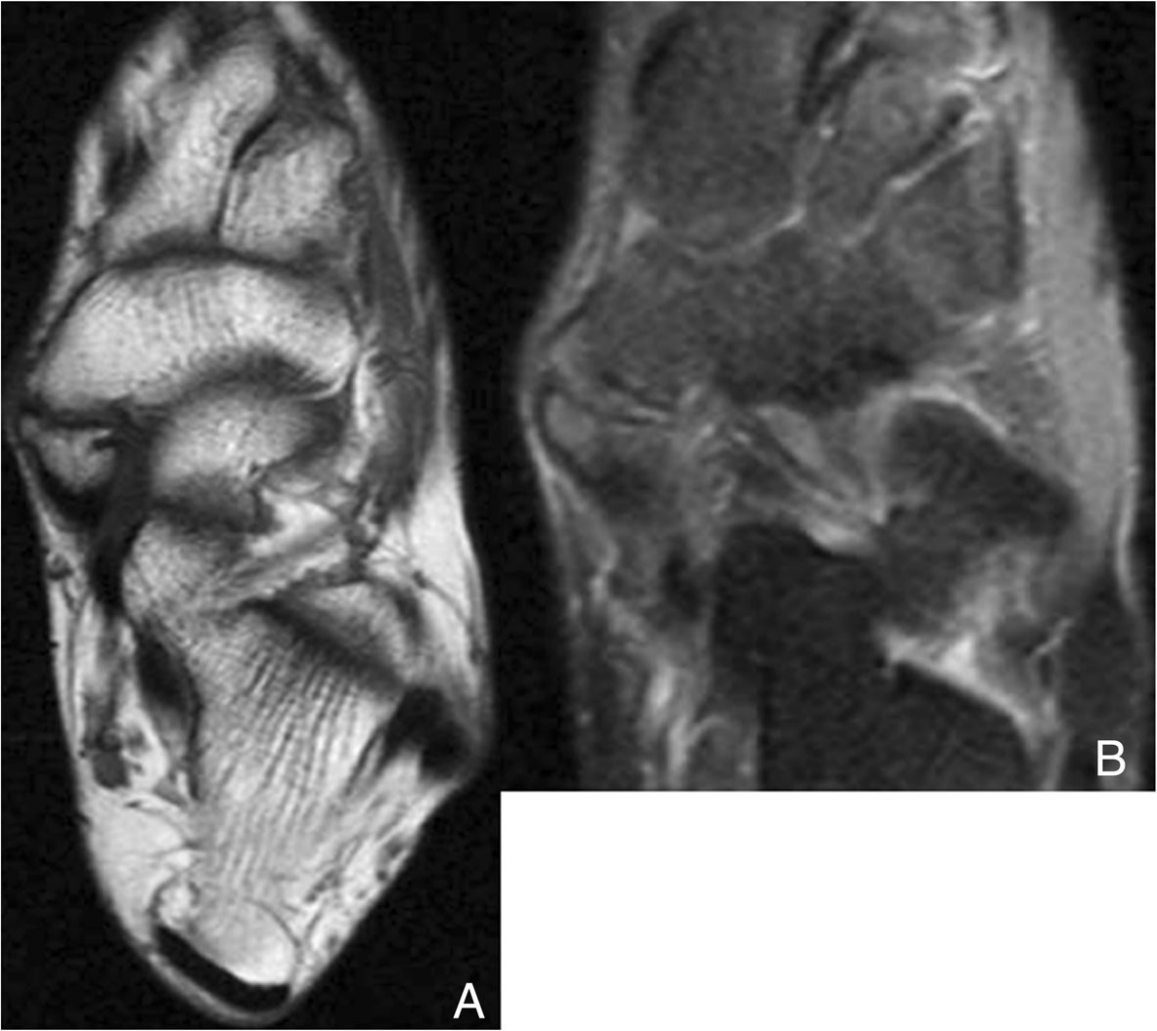
Type II accessory navicular. **a** Axial FSE T1 in a 37-year-old woman with history of tibialis posterior insufficiency. There is a type II accessory navicular, with irregularity in the articular facets of the synchondrosis, indicative of abnormal mobility / mechanical overload. **b** Axial fast spin echo proton density (FSE PD) fat sat in the same patient demonstrates increased signal intensity in keeping with edema in both aspects of the synchondrosis, indicating shearing and stress in the joint, which is associated to the tibialis posterior insufficiency

Repetitive shearing forces in the synchondrosis may cause disruption, which may also be followed by flat-foot deformity (Fig. 4). The accessory navicular can also suffer osteonecrosis [10].

Patients will present with foot pain over the medial aspect of the midfoot in every case, and may show features of flat-foot deformity and inability to single-heel lift, in the cases where tibialis posterior loss of function has developed [11].

Radiographs will be able to detect the presence of an accessory ossicle and deformity associated to flat-foot, and occasionally soft tissue swelling in the region. In some cases, the presence of the ossicle and associated pathology in the posterior tibialis insertion and surrounding soft tissues can be demonstrated on ultrasound [12]. Magnetic resonance imaging (MRI) is the preferred imaging tool for accurate diagnosis, and will show bone marrow edema in both aspects of the synchondrosis in the cases of shearing and mechanical stress, and potentially, also associated pathology of the tibialis posterior, such as signs of tenosynovitis, tendinosis, or tear. In the cases of osteonecrosis, bone marrow edema will be present in the ossicle [9, 13, 14].

**Fig. 5:**
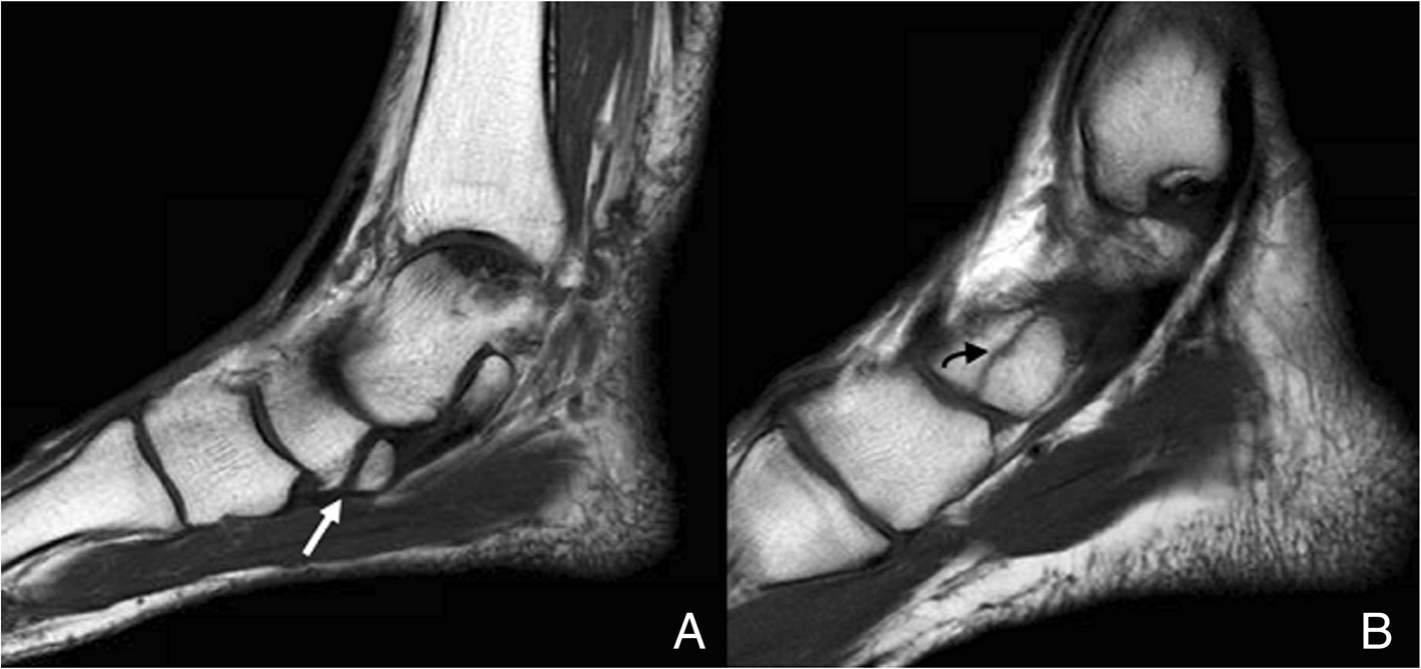
Differential diagnosis type II accessory navicular and navicular fracture. **a** Sagittal FSE T1 showing the incidental finding of a type II accessory navicular (white arrow). Note how the synchondrosis represents a regular line. **b** A 51-year-old man with history of sprain several months ago and persisting pain in the medial aspect of the midfoot. Sagittal FSE T1 demonstrates the sequel of a transverse slightly irregular hypointense line of fracture through the navicular, with no displacement of fragments (curved black arrow)

Differential diagnosis has to be made with avulsion fractures of the tuberosity [7], which result from acute eversion of the foot and increased tension of the posterior tibialis tendon. These may happen in conjunction with impaction fractures of the cuboid, which may help in diagnosis [15]. A background of trauma and an irregular instead of a smooth separation line from the tubercle will suggest fracture (Fig. 5). The ossicle is normally well corticated with smooth contours, and bilateral. Clinically, and sometimes radiologically, a complete disruption of the synchondrosis with avulsion of the ossicle can be very similar to a fracture [16].

### Os supranaviculare

The os supranaviculare is also known as the os talonaviculares dorsalis, talonavicular ossicle, or Pirie’s bone. It can be found in the dorsal aspect of the talonavicular joint.

**Fig. 6:**
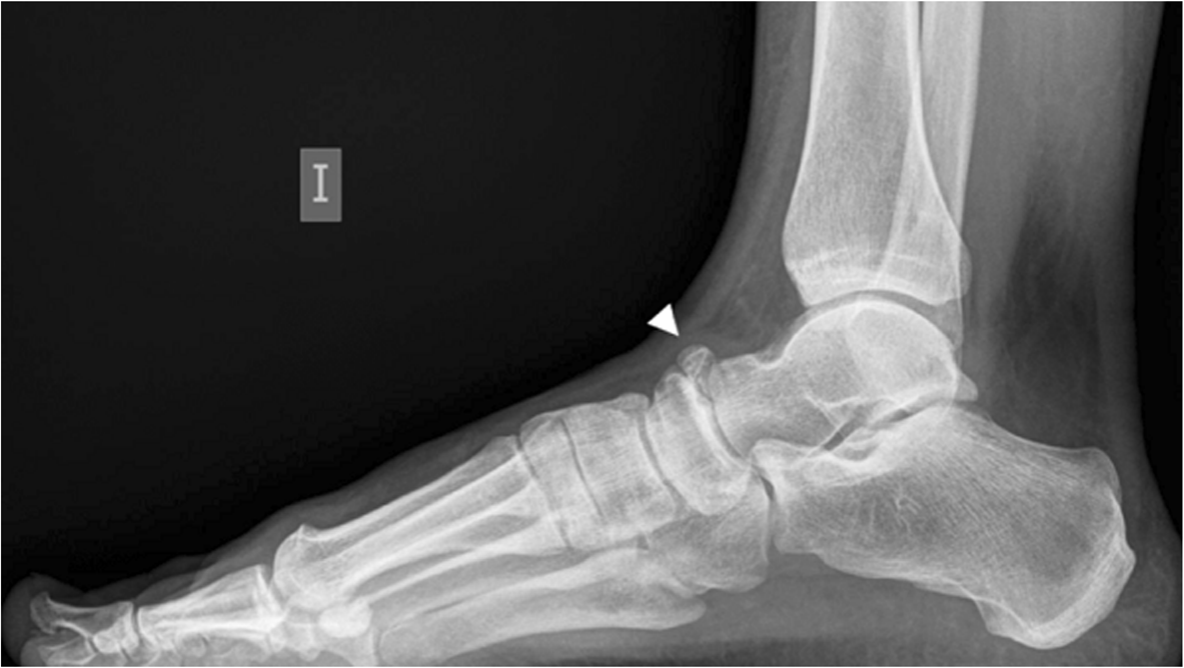
Os supranaviculare. Example of an incidental finding of an os supranaviculare (arrowhead) on a 39-year-old woman, referred with the suspicion of sesamoiditis

This is a rare os, with a prevalence that has been estimated as 1%, and usually asymptomatic [2]. In some cases, it is fused with the navicular, to form a spur that has no clinical significance **(**Fig. 6**)**.

**Fig. 7:**
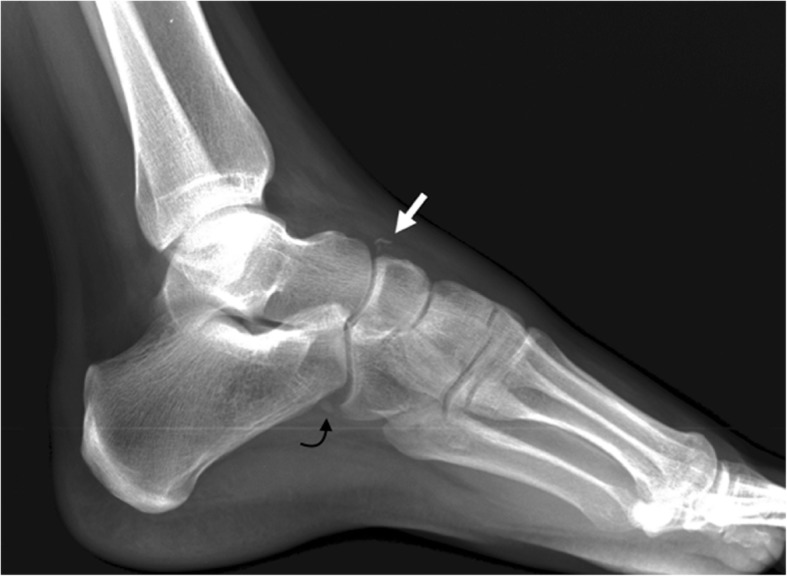
Differential diagnosis of os supranaviculare. The differential diagnosis has to be established with an avulsion fracture of the talo navicular joint capsule, as seen in this lateral radiograph (white arrow). Note the mild swelling of the soft tissues associated and the more linear configuration of the fragment, compared to the more triangular shape of the ossicle. Incidental note os peroneum (curved black arrow)

Differential diagnosis has to be established with avulsion fractures of the capsule of the talonavicular joint, which happen typically in middle-aged women and are related to the use of high heels [15]. Fractures of the capsule may associate fractures in the dorsal aspect of the talar head, which may help in the differential. History of trauma and development of pain and swelling in the region, and a more linear morphology favors fracture over ossicle [7] (Fig. 7).

### Os peroneum

The os peroneum represents a sesamoid bone included within the peroneus longus tendon, normally located at the level of the calcaneo-cuboid joint, just proximal to the peroneal groove of the cuboid [17]. Occasionally, it may lie in the plantar aspect of the cuboid and articulate with it.

The os peroneum, as an anatomical structure, is thought to be present in everyone, at least in its cartilaginous form, but accurate prevalence remains unclear [18]. There is controversy on whether this has an embryonic development or whether it constitutes a stress response due to the configuration and course of the peroneus longus tendon [19].

In its ossified form prevalence has been estimated from 3 to 26% [17, 20].

The os peroneum is bipartite or multipartite in up to 30% of cases, and bilateral in 60% [17]. It is best seen in oblique radiographs of the foot.

The presence of an os peroneum may be linked to pathology, known as “painful os peroneum syndrome”, which can be acute or present as an overuse chronic condition. The syndrome consists of pain and swelling following the course of the peroneus longus tendon between the malleolus and cuboid, and lateral pain with resisted plantar flexion of the foot [3].

Among the acute causes, ankle sprain is a common one, through forceful contraction of the peroneus longus tendon during forced supination or dorsiflexion of the foot. Fractures of the ossicle and tears of the peroneus tendon longus are typical of acute presentation.

In a recent review, Bianchi et al. have proposed a three-type classification for peroneus longus tears in the context of the presence of an os peroneum, depending on whether the tear occurs proximally to the ossicle (type I), through the ossicle as a true fracture (type II), or distal to the ossicle (type III).

In fractures through the ossicle (type II), if fragments are not displaced, differential has to be made with a multipartite ossicle, through the rounder appearance of the components of the ossicle as opposed to sharp edges of fracture fragments. Serial radiographs to assess for progressive displacement, that is frequent due to the tensile force of the peroneus longus, and contralateral comparison of position in the case of bilateral ossicles may also help.

When tears happen distally to the ossicle (type III), this may migrate proximally and be superimposed to the calcaneus and therefore difficult to detect on lateral radiographs, or be considered an unusually located os trigonum, or be mistaken with an os subfibulare on computed tomography (CT) [19].

Chronic painful os peroneum syndrome is in general more often described. It is due to repeated friction of the os against the cuboid, stress fractures, local impingement in the cases of hypertrophic os peroneum, or repetitive sports activities with a component of hypersupination, and the existence of partial tears [17, 21, 22].

**Fig. 8:**
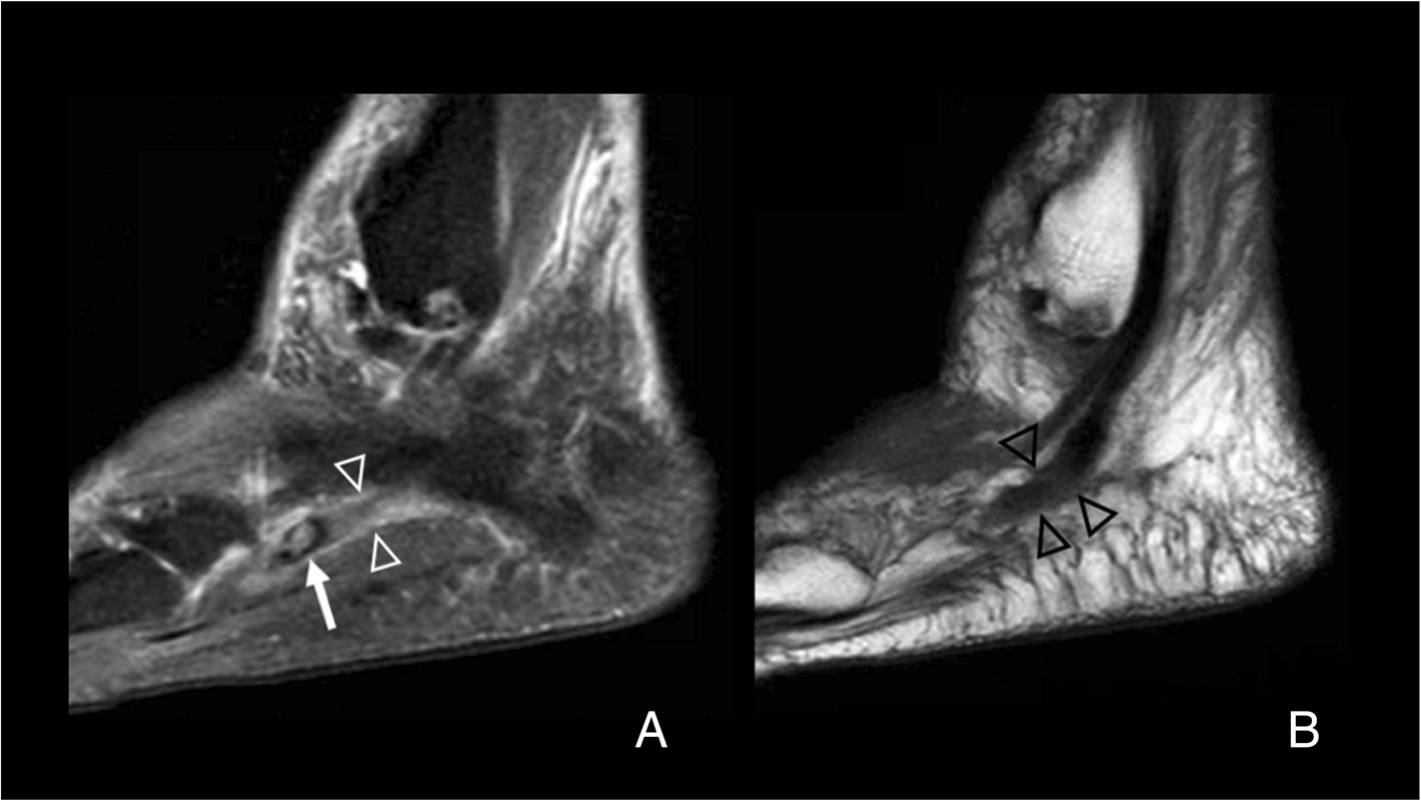
Symptomatic os peroneum. **a** A 61-year-old woman, referred for lateral ankle pain. Sagittal proton density spectral attenuation inversion recovery (PD SPAIR). High signal intensity is present in the os peroneum (white arrow), in keeping with bone marrow edema. The peroneus longus tendon appears hyperintense (arrowheads). **b** On FSE T1, in a slightly more lateral plane, the increased signal intensity in the peroneus longus tendon is also evident (black arrowheads), proximal to the os peroneum, in keeping with tendinopathy

Pathology of the os can be detected with all imaging modalities. Radiographs are ideal for the assessment of shape, contours, and location of the ossicle, and similarly is CT. Ultrasound (US) can demonstrate pathology of the peroneus longus and grade it, and detect fractures, especially if there is displacement. MRI is not ideal for the evaluation of shape and contours; however, it will depict very clearly alterations on bone marrow signal and demonstrate pathology in the tendon [3, 19, 23] **(**Fig. 8**)**.

### Rare bone variants

Bipartite bones can be present in the midfoot. The most frequently involved bone is the medial cuneiform. Its incidence is estimated in between 0.3 and 2.4% [24].

**Fig. 9:**
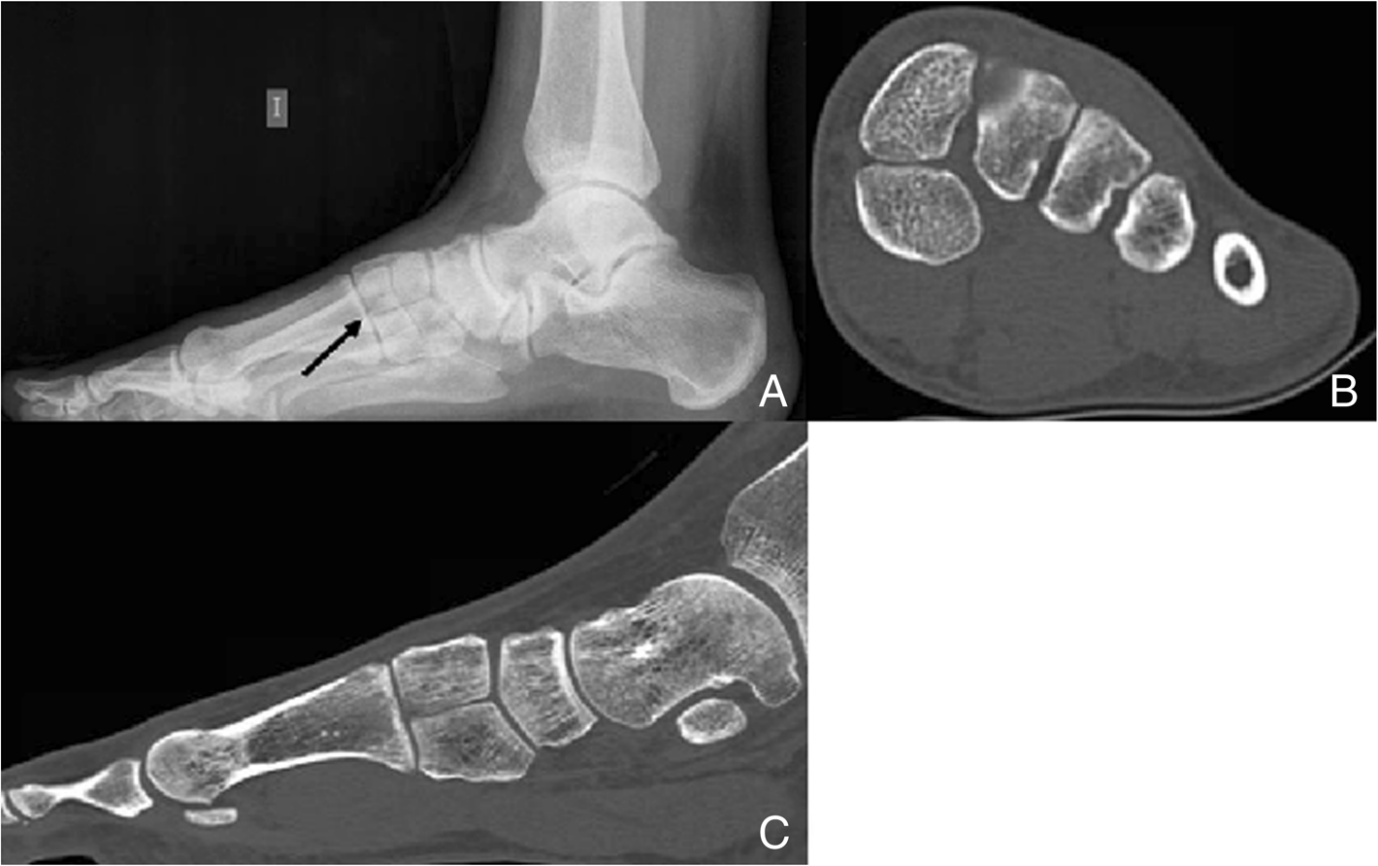
Bipartite medial cuneiform. A 32-year-old man, on work up for left flat foot. Bilateral foot radiographs were taken. **a** Left lateral view demonstrates a bipartite cuneiform (finding was bilateral but only right foot shown). The black arrow marks the synchondrosis in between medial cuneiform components. **b** CT coronal reconstructions show a slightly larger plantar cuneiform. **c** Sagittal CT reconstruction shows that the addition of the volume of the two bones is larger than a normal medial cuneiform would be

A bipartite medial cuneiform has a plantar and a dorsal component, that articulate through a synchondrosis, which is subject to degeneration and overuse syndromes (Fig. 9).

The common differential will be a fracture through the medial cuneiform. Rounder margins, and an added volume greater that the normal volume of a medial cuneiform suggest bipartite configuration over fracture [25].

A rare variant is the os cuboideum secundarium, with the potential of mimicking a mass on MRI. Very few case reports exist in the literature [26–28].

Another very rare variant is the os intercuneiform, with a frequency estimated as 0.026% in large anatomical series [29].

## Forefoot

For the purpose of the description, the forefoot is defined as the region distal to Lisfranc joint (tarso-metatarsal joint)

**Fig. 10:**
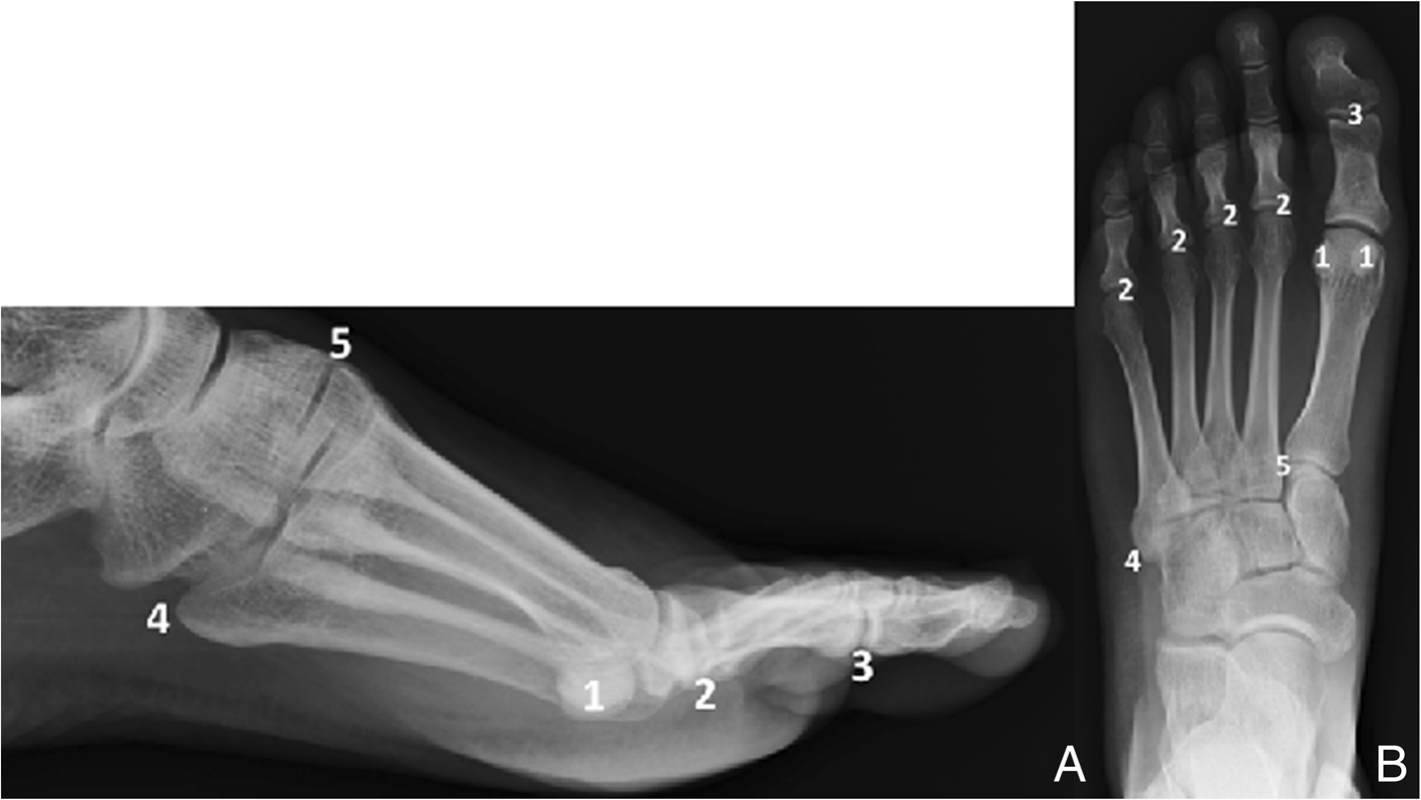
Diagram of the location of the most common accessory bones of the forefoot. **a** Lateral and (**b**) AP projection of the forefoot. 1—Hallux sesamoid, 2—lesser metatarsal sesamoids, 3—interphalangeal joint sesamoids, 4—os vesalianum, 5—os intermetatarseum

- Ossicles (Fig. 10) (Table 2)

**Table 2:** Prevalence, clinical significance, and differential diagnosis of the most common types of variants and accessory ossicles in the forefoot

Ossicle	Prevalence		Clinical significance	Differential diagnosis
Hallucal sesamoids	Multipartite (gen medial)	2.7–33.5% bilateral—22–85%	Potential sesamoiditis (osteoarthritis—osteonecrosis)	Sesamoid fracture (multipartite)
	Absence	rare		
Lesser metatarsal sesamoids	2nd digit	0.4%	Asymptomatic (Infection from surrounding soft tissues)	–
	3rd digit	0.2%		
	4th digit	0.1%		
	5th digit	4.3%		
Interphalangeal joint sesamoids	2–13% ossified 73% nodule in cadaver series		Interposition in joint dislocation. Limitation to joint mobility and painful callosity have been reported	–
Os vesalianum	0.1 to 1%		Very rarely a source of pathology Painful conditions similar to the os peroneum syndrome have been reported	Avulsion fractures of the apophysis and base of the fifth metatarsal
Os intermetatarseum	1.2–10%		Pain on palpation of the dorsum of the foot (superficial and deep peroneal nerves compression)	Small fractures of the base of the second metatarsal in Lisfranc fracture—dislocations

### Variations of the hallucal sesamoids

The hallux sesamoids are located within the medial and lateral slips of the flexor hallucis brevis tendon. They lie at the level of the head of the first metatarsal, and are separated from each other by a small bony ridge, called the crista, located in the plantar aspect of the metatarsal head. The hallux sesamoids articulate with the plantar aspect of the metatarsal head. Their deep surfaces are covered by hyaline cartilage and included within the capsule of the joint, constituting a synovial joint [30]. They are embedded within the plantar plate, stabilized by the medial and lateral capsular ligament and phalangiosesamoid ligaments, and connected with each other through the intersesamoid ligament. Besides from this, the medial sesamoid is further stabilized by fibers of the abductor hallucis tendon, and the lateral by the adductor hallucis tendon [3].

The sesamoids increase the mechanical potential of the hallux flexors, besides from protecting the tendon that runs in between them and acting as shock absorbers for the first metatarsal head. They are therefore paramount in the biomechanics of the first metacarpal joint [24].

**Fig. 11:**
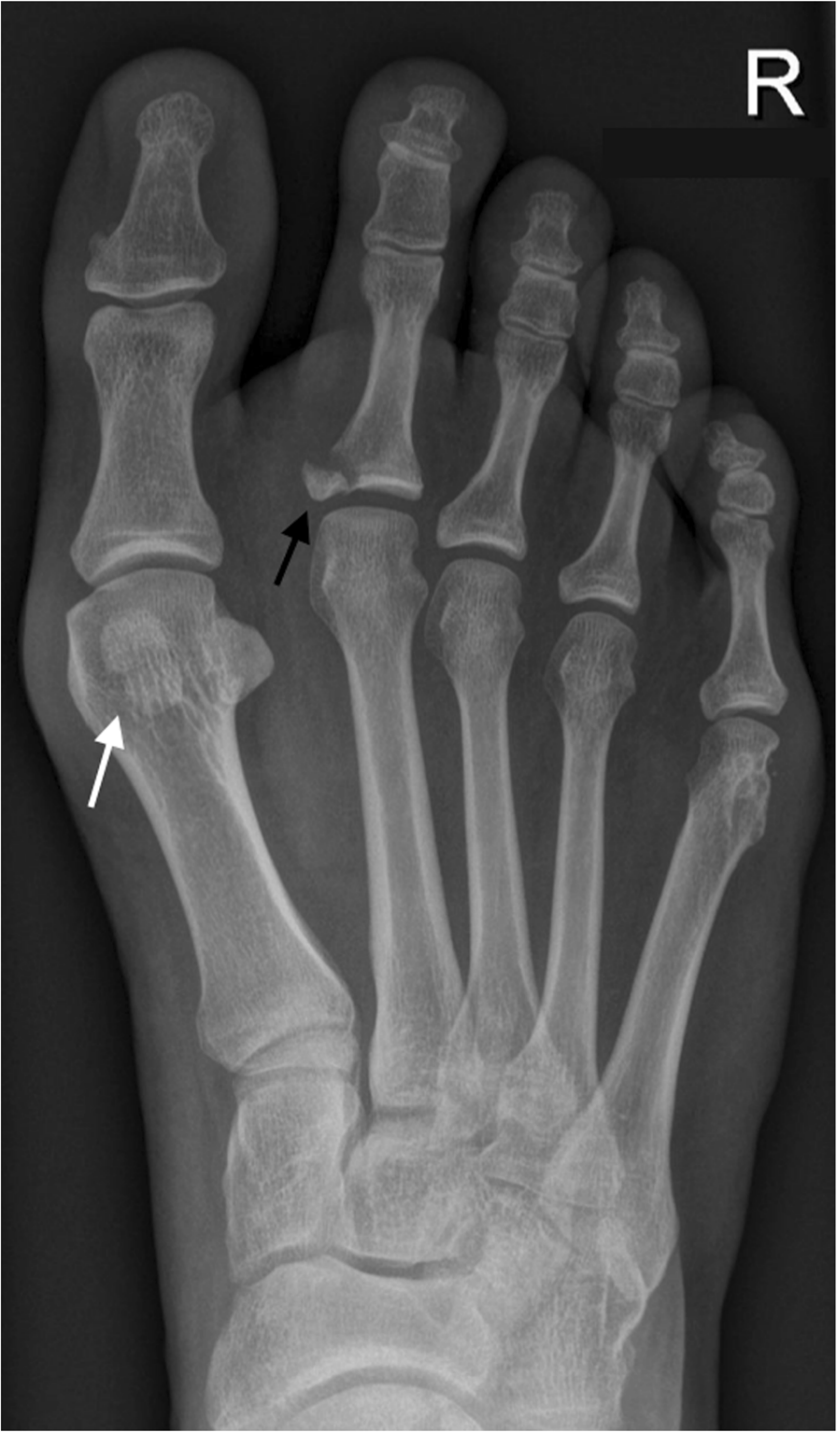
A 41-year-old woman referred after kite surfing trauma. Incidental finding of a medial bipartite sesamoid (white arrow). Note the smooth, rounded contours of the bipartite components. The added volume of the bipartite components adds to a larger volume than a single sesamoid. Note mild hallux valgus deformity and a fracture of the base of the proximal phalanx in the second toe (black arrow)

Incidence of multipartite sesamoids has been reported from 2.7 to 33.5%. It is more common to find medial bipartite sesamoids than lateral sesamoids. Medial bipartite sesamoids can be bilateral in a frequency ranging from 22 to 85% [3] (Fig. 11). Congenital absence of a sesamoid has been described, but is extremely unusual [31].

Sesamoids are subject to the same pathological conditions as any other synovial joint, with the possibility of degenerative change, infective and inflammatory conditions, and osteonecrosis.

Sesamoiditis is a term that clinically represents pain in the sesamoid region.

**Fig. 12:**
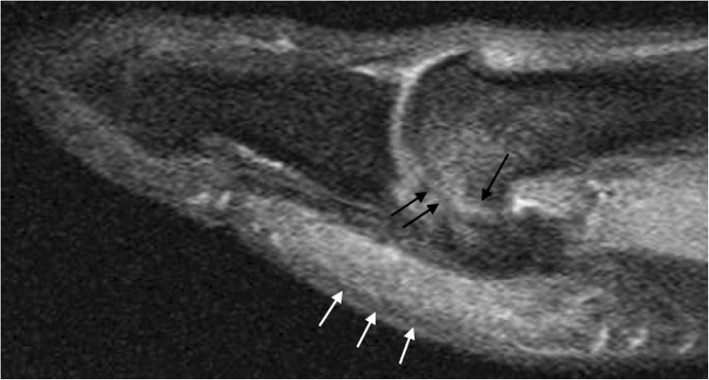
Sesamoiditis. A 39-year-old woman, referred with the suspicion of sesamoiditis. Sagittal Fast FSE PD fat sat demonstrates increased signal intensity in both aspects of the sesamoid—metatarsal joint, with loss of cartilage and narrowing of the joint space (black arrows), as well as associated increased signal intensity in the subcutaneous tissue fat in the plantar aspect (white arrows). Findings are in keeping with degenerative change in joint, but there is added adventitial bursitis reaction

This can be caused by multiple different conditions, the most common one being osteoarthritic changes in the joint with the metatarsal head. Typically, changes in both aspects of the joint will be present in these cases in the different imaging modalities. The joint will show the characteristic features of narrowing, subchondral sclerosis, and development of geodes and osteophytes. On MRI, there will be cartilage loss and evolving subchondral change, with bone marrow edema as an early sign (Fig. 12).

The fact that changes are present in both aspects of the joint is useful in distinguishing degenerative change from other causes of sesamoid pain, such as a stress reaction, where MRI findings will be similar but only appear in the sesamoid. The sesamoid may eventually end up fragmented. In other causes for chronic sesamoid pain, such as osteonecrosis, findings on MRI will resemble a stress reaction as a start, and will overlap with osteoarthritis changes as collapse progresses and mechanical alteration develops [24].

Repetitive and exaggerated axial load in plantar flexion, which is typical of activities such as ballet or running are typical causes for sesamoiditis. Tendinosis and capsular inflammation can also develop and be a cause for chronic pain in the region [32].

**Fig. 13:**
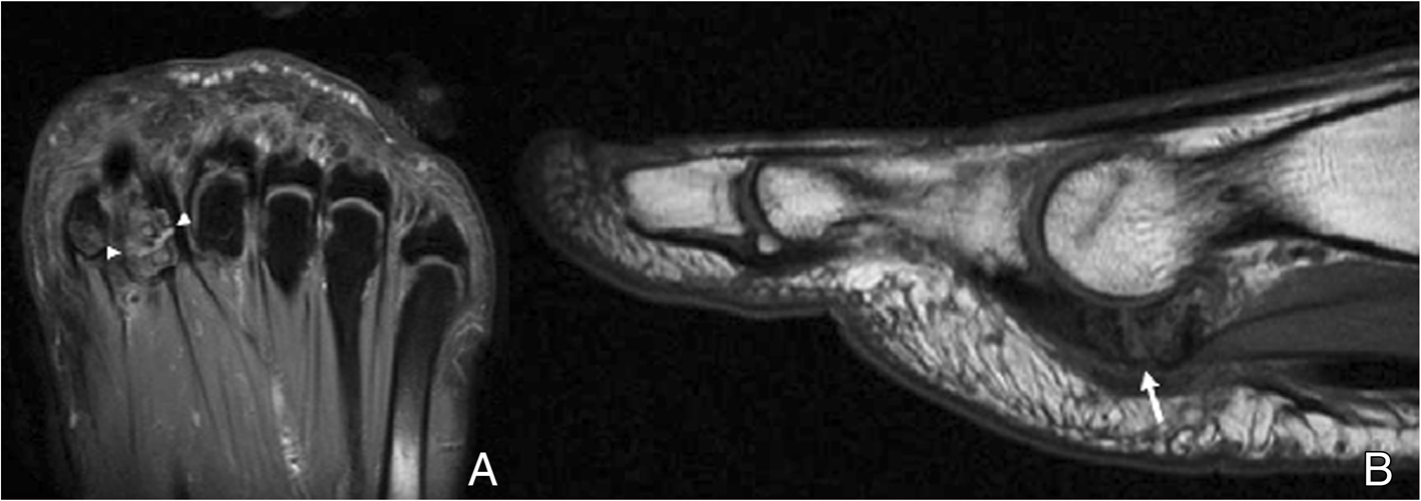
Lateral sesamoid fracture. A 35-year-old man with pain over plantar aspect of foot after trauma. **a** Axial FSE PD fat sat demonstrates a fracture of the lateral sesamoid, with slight separation of fragments, and a band of interposed fluid (white arrowheads). **b** Sagittal FSE T1 demonstrates hypointensity in the fracture fragments. The fracture line is visible, slightly more hyperintense than the adjacent fragments (white arrow)

It is important to consider that the bipartite sesamoid configuration can present superimposed pathology, and in cases in which clinical symptoms suggest a fracture and there is no previous knowledge of the variant, distinguishing in between other causes for pain and a fracture can be challenging. In the case of fracture, there is usually a sharp parting line, with interposed fluid, and the fragments fit together (Fig. 13). On MRI, there will be bone marrow edema and edema in the surrounding soft tissues.

In the case of bipartition, the components are usually rounder and would configure a greater than normal sesamoid if their sizes were added. On MRI, there could be edema in the cases of stress reaction or mechanical overload [24].

### Lesser metatarsal sesamoids

Sesamoids adjacent to the second through to fifth metatarsal heads are embedded in the plantar aspect of the joint capsule and may be bipartite or multipartite.

The most frequent one is the adjacent to the fifth metatarsal head one, with a prevalence of up to 4.3%, followed by 0.4% at the second, 0.2% at the third, and 0.1% at the fourth [33].

**Fig. 14:**
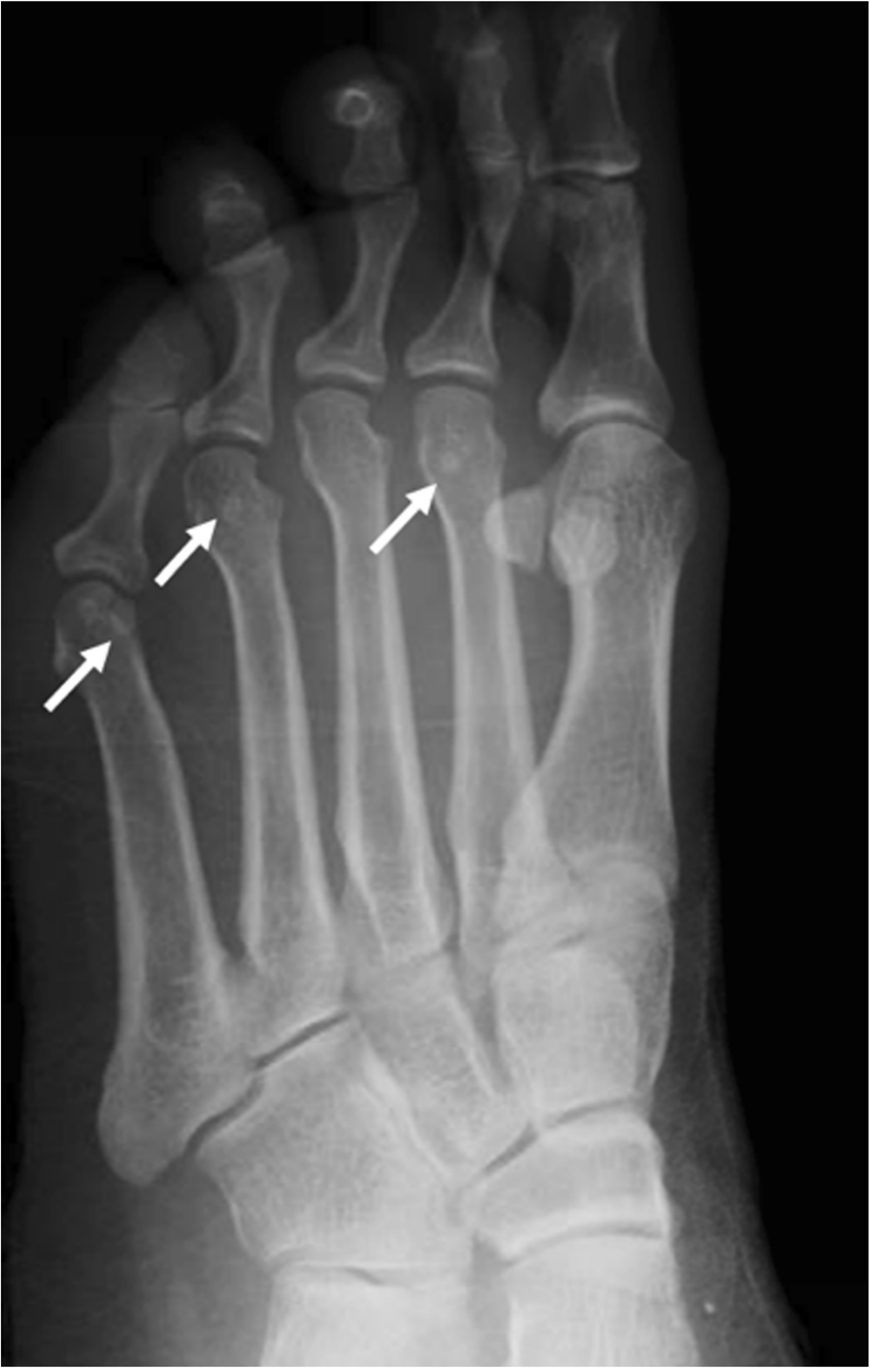
Lesser metatarsal sesamoids. Oblique radiograph on a 42-year-old woman, referred after trauma of the ankle. Incidental finding of multiple lesser sesamoids in the fifth, fourth, and second metatarso-phalangeal joints (white arrows). The fifth is the most frequently found one. Lesser sesamoid pathology is very rare

Pathology is very rare. Infection from direct spread from adjacent soft tissue is a possibility [6] (Fig. 14).

### Interphalangeal joint sesamoids

**Fig. 15:**
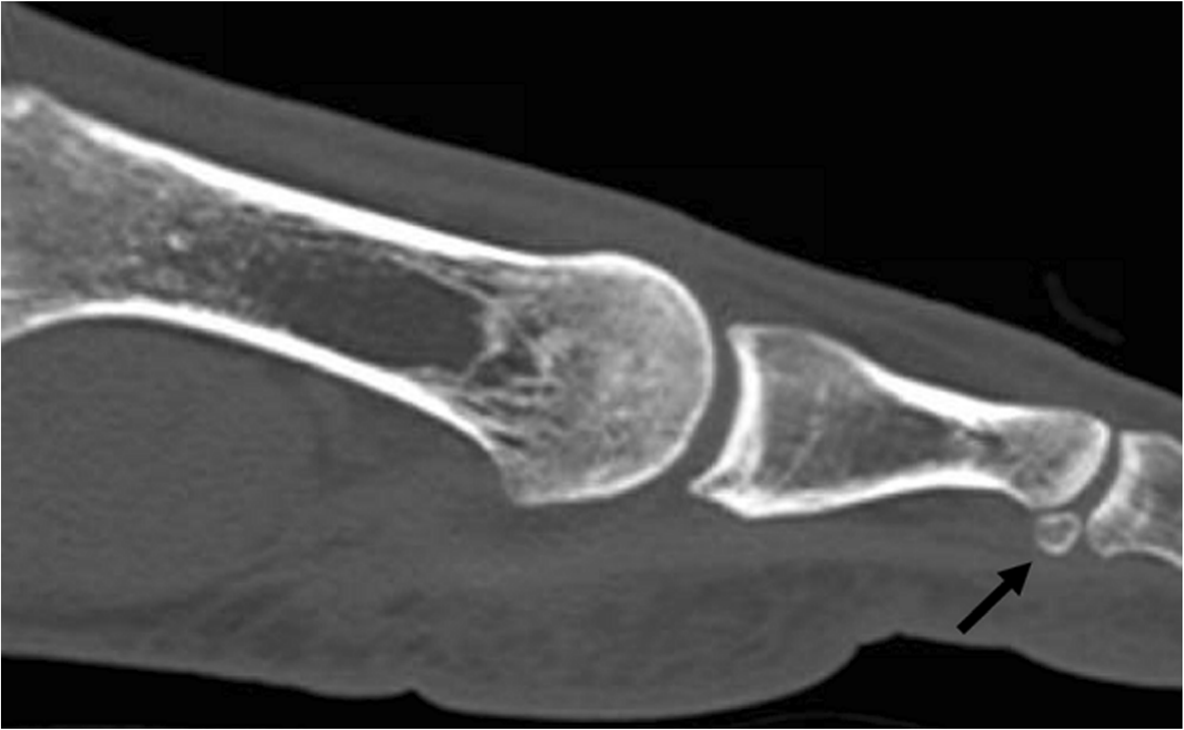
Interphalangeal joint sesamoid. A 33-year-old man referred for flat foot. Incidental finding of an interphalangeal joint sesamoid. Sagittal CT reconstruction better demonstrates its position with respect to the interphalangeal joint (black arrow). A potential complication arises if there is a phalangeal dislocation and the ossicle displaces into the joint space, blocking reduction

They are located at the interphalangeal aspect of the interphalangeal joint of the great toe. They are embedded within the joint capsule. Prevalence in its ossified form has been reported as 2–13% [33]; however, a nodule in the joint has been identified in 73% in a cadaver series [34] (Fig. 15).

Pathology linked to them is rare, but limitations to joint mobility and painful callosity have been reported.

A potential complication derived from the presence of an interphalangeal sesamoid is its interposition in the case of interphalangeal joint dislocation, making it impossible to reduce [35, 36].

### Os vesalianum

The os vesalianum is located proximal to the apophysis of the fifth metatarsal, within the peroneus brevis tendon.

Its prevalence has been estimated as 0.1 to 1% [6].

This accessory ossicle is very rarely a source of pathology, but painful conditions similar to the os peroneum syndrome have been reported [37, 38].

The os vesalianum needs to be distinguished from the normal ossification center of the tuberosity of the fifth metatarsal in children, which is normally parallel to the shaft.

This ossicle is located more distally than the os peroneum (which is normally projected over or adjacent to the calcaneo-cuboid joint) and can be identified in AP or lateral views. Differently to the os peroneum, which is located within the peroneus longus tendon, the os vesalianum is located within the peroneus brevis tendon.

**Fig. 16:**
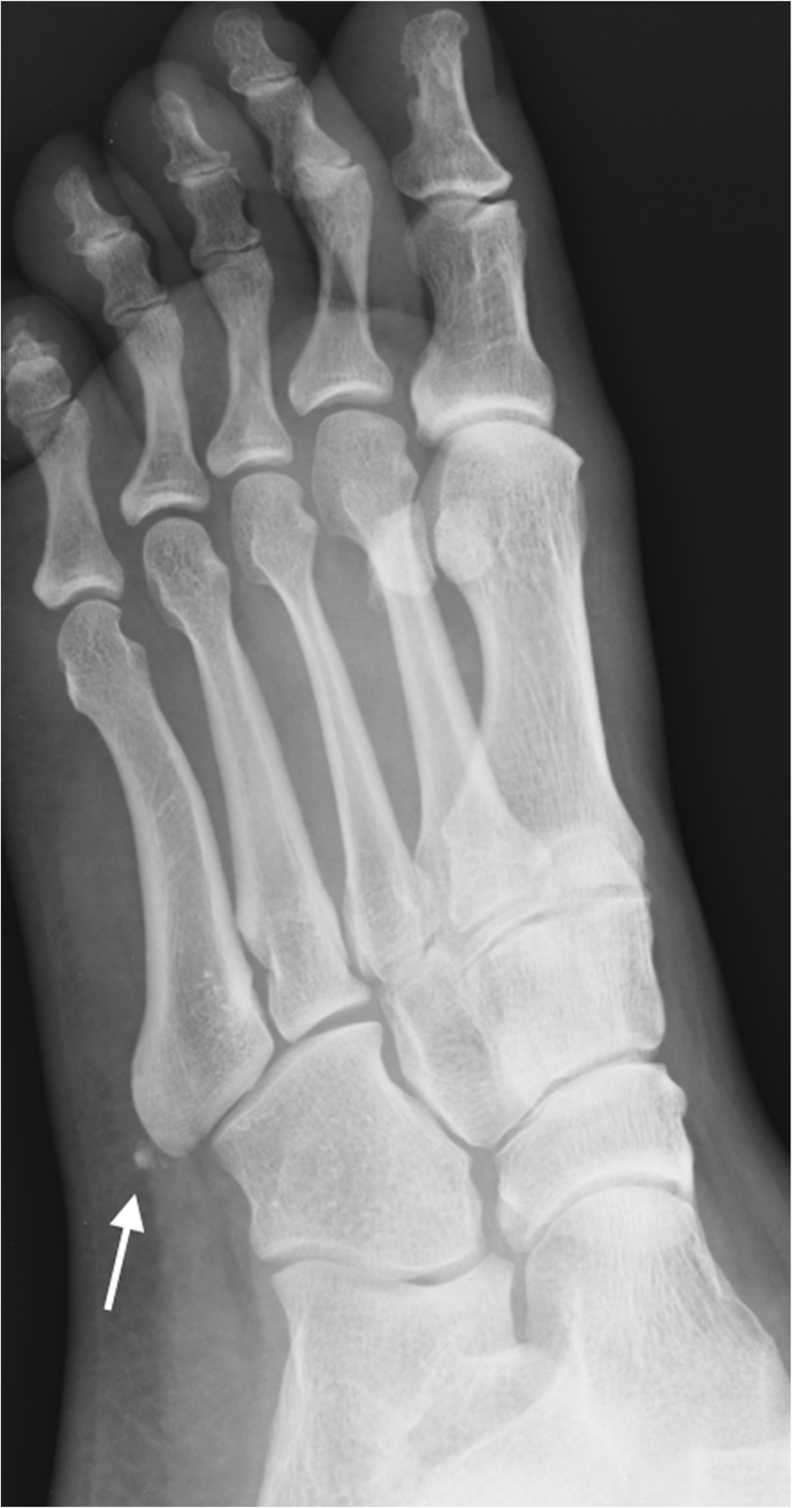
Os vesalianum. Oblique radiograph. Incidental finding of a small os vesalianum (white arrow). These very rarely constitute a source of pathology

**Fig. 17:**
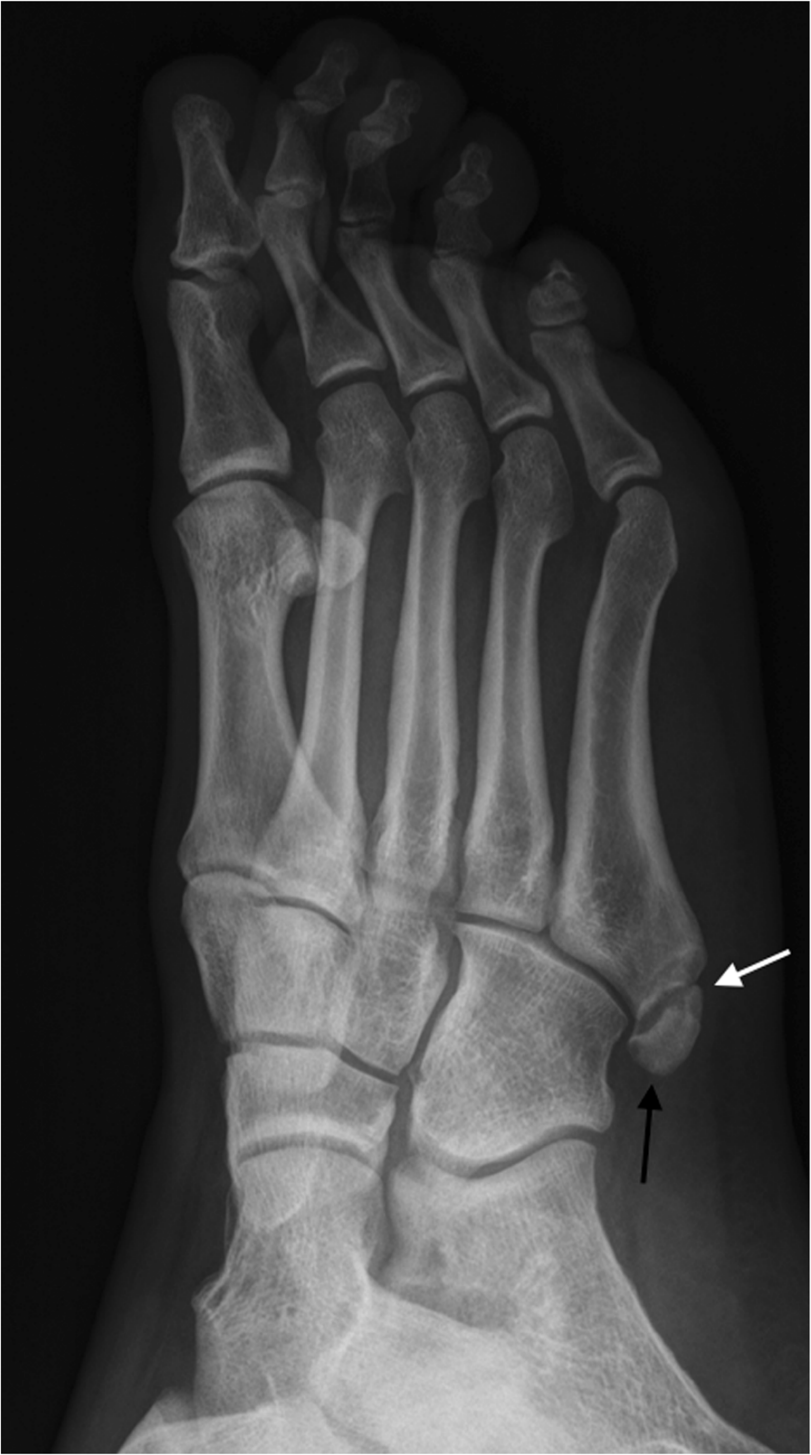
Differential of os vesalianum. Oblique radiograph. Old non-united fracture fragment of the apophysis of the fifth metatarsal (black arrow). The fracture line is visible (white arrow). This finding, in some occasions, can resemble an os vesalianum

Differential diagnosis has to be made with avulsion fractures of the apophysis and base of the fifth metatarsal. These are normally transverse and there is a history of inversion injury [7] (Figs. 16 and 17).

### Os intermetatarseum

**Fig. 18:**
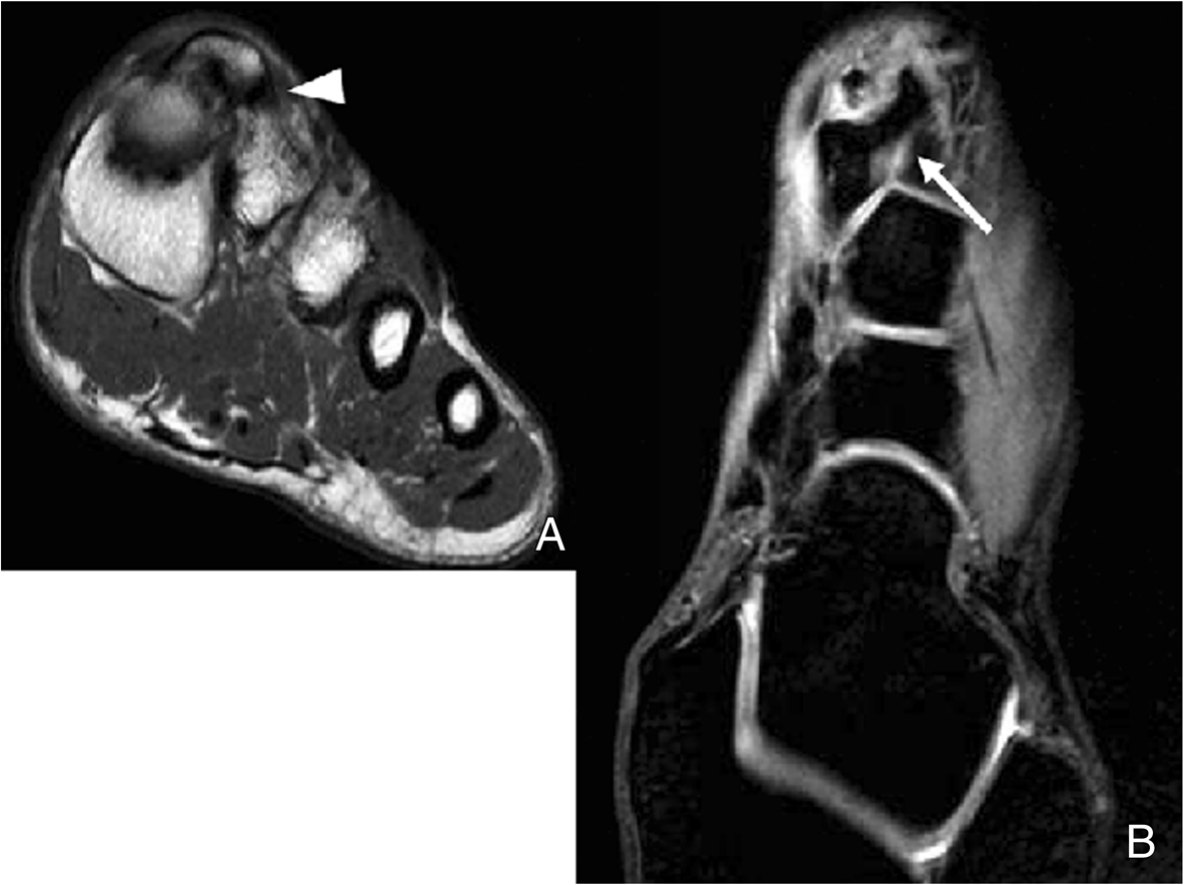
Os intermetatarseum. A 36-year-old man, investigated for pain in the Achilles. Radiographs demonstrate a spur projection in the dorsum of the foot, overlying the base of the metatarsal (not shown). **a** Coronal FSE T1 demonstrates how this articulates with the base of the second metatarsal, with a synchondrosis (white arrowhead). **b** Axial three-dimensional gradient echo water selective/fluid (WATSf) demonstrates this exostosis arises from the base of the first metatarsal, and extends over the joint with the base of the second metatarsal (white arrow). These ossicles rarely represent a cause or pathology, unless compression irritates the superficial and deep peroneal nerves

The os intermetatarseum is located dorsally between the medial cuneiform and the base of the first and second metatarsals. It may exist separated from these bones, articulate with them with a synovial articulation or be fused to any of them and present as a spur projection [10, 39]. The os can be round or spindle shaped (Fig. 18).

Prevalence has been estimated as 1.2–10% [7]; however, there is still controversy about its origin and real prevalence.

The os can suffer fracture or be related to painful conditions [40].

The symptomatic os intermetatarseum causes pain on palpation of the dorsum of the foot, because superficial and deep peroneal nerves are compressed [40, 41].

Differential diagnosis has to be made with small fractures of the base of the second metatarsal in Lisfranc fracture—dislocations. These fractures usually occur as a result of exaggerated plantar flexion and rotation, normally in a high-energy trauma setting, and associate malalignment and soft tissue swelling [6].

b. Soft tissues of the midfoot and forefoot (Table 3)

**Table 3:** Origin, insertion, and clinical significance of rare accessory muscles around the mid and forefoot

Muscle	Origin	Insertion	Clinical significance
Accessory plantar muscle belly to the adductor hallucis	Fourth metatarsal	Oblique and transverse heads of the adductor hallucis	Transposition to alleviate symptoms of hallux valgus
Variant of the flexor digitorum accesorius or quadratus plantae	Calcaneus	Lateral aspect of the flexor digitorum longus tendon	Tarsal tunnel syndrome
Accessory flexor digiti minimi pedis (duplicated variant)	Tibialis posterior	Middle phalanx of the fifth digit	Graft—transposition

Variations related to attachments and slips of muscular structures can be found in the midfoot and forefoot, but these are usually asymptomatic.

Accessory muscles are less common than in the ankle, and also frequently asymptomatic. They may cause a mass effect and result in compressive neuropathies in rare occasions.

Some of accessory muscles with potential implications have been listed in the literature. An accessory plantar muscle belly to the adductor hallucis has been described, arising from the fourth metatarsal and inserting with the normally configured oblique and transverse heads of the adductor hallucis [42]. Given the adductor hallucis can be used in transposition to alleviate symptoms of hallux valgus and in reconstructive surgeries to cover defects, variations in its normal configuration and anatomy can be relevant.

A variant of the flexor digitorum accesorius or quadratus plantae has been associated with tarsal tunnel syndrome. The traditionally described configuration of the muscle arises from the calcaneus and inserts onto the lateral aspect of the flexor digitorum longus tendon. In a cadaveric study, the muscle was found to have high variability in its configuration, with more variability in the origin of its medial head, which in 80% of the cases was seen to extend into the tarsal tunnel. The lateral head was seen to be less variable and almost always have an aponeurotic origin [43].

A rare accessory flexor digiti minimi pedis arising from the tibialis posterior and inserting on the middle phalanx of the fifth toe has also been described [44], as well as duplication of the flexor digiti minimi pedis. These can also be used for grafting—transposition [42].

## Conclusion

This review has illustrated the imaging findings related to the presence of accessory ossicles and muscles in the midfoot and forefoot through different techniques, and the potential clinical implications related to their existence, highlighting the importance of each technique in the diagnosis and assessment of related pathology.

Most accessory ossicles and sesamoids will represent an incidental finding on radiographs. Accessory muscles can occasionally represent an incidental finding on radiographs, but are mainly incidentally noted on MRI or CT.

In the cases where pathology in relation of the presence of these structures is suspected, detailed clinical correlation and careful assessment with MRI and CT will play an important role.

It is useful for the radiologist to be familiar with the characteristics of these anatomical variants to avoid misdiagnosis.

## Abbreviations

CT: Computed tomography
FSE PD: Fast spin echo proton density
FSE T1: Fast spin echo T1
MRI: Magnetic resonance imaging
PD SPAIR: Proton density spectral attenuation inversion recovery
US: Ultrasound
WATSf: Water selective/fluid
